# Effect of Soybean Oil Supplementation on Milk Production, Digestibility, and Metabolism in Dairy Goats under Thermoneutral and Heat Stress Conditions

**DOI:** 10.3390/ani11020350

**Published:** 2021-01-30

**Authors:** Soufiane Hamzaoui, Gerardo Caja, Xavier Such, Elena Albanell, Ahmed A. K. Salama

**Affiliations:** Group of Research in Ruminants (G2R), Animal and Food Science Department, Universitat Autònoma de Barcelona, Bellaterra, 08193 Barcelona, Spain; sofianina2000@yahoo.fr (S.H.); gerardo.caja@uab.cat (G.C.); xavier.such@uab.cat (X.S.); elena.albanell@uab.cat (E.A.)

**Keywords:** heat load, milk fatty acids, lactation, digestion, blood metabolites

## Abstract

**Simple Summary:**

Heat stress (HS) not only reduces milk yield but also depresses its contents of fat and protein, which might negatively impact cheese making. Dietary supplementation with soybean oil (SBO) could increase milk fat and improve milk fatty acid (FA) profiles in dairy goats. In the present study dairy goats were exposed to thermoneutral (TN; 15 to 20 °C) or HS (12 h/d at 37 °C and 12 h/d at 30 °C) conditions. In each ambient temperature, goats were fed a control diet (CON) or the same diet supplemented with SBO. Goats in HS suffered depressed feed intake and milk production, but they had greater digestibility coefficients compared to TN goats. Regardless of the HS treatment, goats supplemented with SBO produced milk with greater contents of fat, monounsaturated FA, and conjugated linoleic acid, without any negative effects on milk protein content. In conclusion, dietary supplementation with soybean oil was a useful strategy to increase milk fat and improve its fatty acid profile. Both TN and HS goats responded to soybean oil supplementation similarly since the interaction between soybean oil supplementation and temperature treatment was not significant.

**Abstract:**

In a previous work, we observed that heat-stressed goats suffer reductions in milk yield and its contents of fat and protein. Supplementation with soybean oil (SBO) may be a useful strategy to enhance milk quality. In total, eight multiparous Murciano–Granadina dairy goats (42.8 ± 1.3 kg body weight; 99 ± 1 days of lactation) were used in a replicated 4 × 4 Latin square design with four periods; 21 d each (14 d adaptation, 5 d for measurements and 2 d transition between periods). Goats were allocated to one of four treatments in a 2 × 2 factorial arrangement. Factors were no oil (CON) or 4% of soybean oil (SBO), and controlled thermal neutral (TN; 15 to 20 °C) or heat stress (HS; 12 h/d at 37 °C and 12 h/d at 30 °C) conditions. This resulted in four treatment combinations: TN-CON, TN-SBO, HS-CON, and HS-SBO. Compared to TN, HS goats experienced lower (*p* < 0.05) feed intake, body weight, N retention, milk yield, and milk protein and lactose contents. However, goats in HS conditions had greater (*p* < 0.05) digestibility coefficients (+5.1, +5.2, +4.6, +7.0, and +8.9 points for dry matter, organic matter, crude protein, neutral detergent fiber, and acid detergent fiber, respectively) than TN goats. The response to SBO had the same magnitude in TN and HS conditions. Supplementation with SBO had no effects on feed intake, milk yield, or milk protein content. However, SBO supplementation increased (*p* < 0.05) blood non-esterified fatty acids by 50%, milk fat by 29%, and conjugated linoleic acid by 360%. In conclusion, feeding 4% SBO to dairy goats was a useful strategy to increase milk fat and conjugated linoleic acid without any negative effects on intake, milk yield, or milk protein content. These beneficial effects were obtained regardless goats were in TN or HS conditions.

## 1. Introduction

Typically, milk production in dairy animals is negatively affected by heat stress (HS) because dry matter (DM) intake is reduced, and a portion of consumed energy is used to maintain homeothermy (greater maintenance requirements). Consequently, the availability of energy and other nutrients for lactation is less, and lower amount of milk is produced with lower contents of fat and protein [1,2]. Heat stress has also a direct negative effect on mammary cells since cell activity and mRNA abundance of genes related to milk synthesis are depressed by HS [3]. Staples and Thatcher [4] evaluated the relationship between milk composition and environmental temperature in dairy cows, and found that as temperatures increase from 9.4 to 36.1 °C, milk fat and protein contents drop by 14 and 13%, respectively. With regard to dairy goats, HS results in milk yield losses (−4 to −16%) with significant depressions in milk fat (−7 to −15%) and protein (−10 to −13%) contents [5,6,7].

Feeding fat is associated with reduced metabolic heat production per unit of energy fed since fat has much lower heat increment in the rumen compared to starch and fiber [8]. Therefore, fats feeding could have positive effects on the performance of heat-stressed animals. However, reports on the fat supplementation under hot conditions were inconsistent [9,10]. Discrepancy among studies might be related to the fact that excessive ruminally active fat in the diet may impair ruminal fermentation and alter the palatability of the ration [11].

Milk fat is energetically more expensive compared to protein or lactose. Bauman and Currie [12] reported that milk fat yield in dairy cows represents up to 35% of the daily net energy intake. Thus, decreasing the energy needed for milk fatty acids (FA) synthesis (e.g., by supplementing preformed FA) could be an effective way not only to improve milk fat content but also to save energy during HS [13].

Supplementation of dairy goats with soybean oil (SBO) alone [14,15,16] or mixed with fish oil [17] results in increased fat content and conjugated linoleic acid (CLA) in milk. Feeding SBO additionally improves yields of milk and protein in dairy goats when the forage:concentrate ratio in the diet is 63:37 [15]. In these aforementioned studies, goats were under normal environmental conditions (no HS). In the literature, no studies are available to evaluate the effect of SBO under TN and HS simultaneously in dairy goats. We hypothesized that SBO supplementation would increase milk fat and protein yields in heat-stressed dairy goats fed 60:40 forage:concentrate ratio. Using the same animals fed the same diet would allow us to precisely test whether the response to SBO supplementation in terms of milk production, milk FA profile, digestibility, and blood metabolites would differ between TN and HS conditions.

## 2. Materials and Methods

### 2.1. Animals, Treatments, and Management Conditions

Multiparous Murciano–Granadina dairy goats (*n* = 8) with healthy and symmetrical udders were used from the herd of the experimental farm of the Universitat Autònoma de Barcelona. Mean days of lactation, body weight (BW), and milk yield were 98 ± 2, 42.8 ± 1.3, and 2.10 ± 0.03 L/d (mean ± SE) at the start of the experiment, respectively. Goats were kept in metabolic cages (1.7 × 0.6 m) with plastic slats and were used in a replicated 4 × 4 Latin square design with 4 periods; 21 days each (14 days adaptation, 5 days for measurements and 2 days transition between periods). Goats were allocated to one of 4 treatments in a 2 × 2 factorial arrangement. Factors were control (CON) without supplementation or 4% of soybean oil (SBO; Gustav Heess, Barcelona, Spain), and thermoneutral (TN; 15 to 20 °C and 40 to 65% relative humidity throughout the day) or heat stress (HS; from 09:00 to 21:00 h at 37 °C and from 21:00 to 09:00 h at 30 °C with 40 ± 5% relative humidity). Goats were maintained under the corresponding treatment throughout the 14 days of adaptation and the 5 days of measurements in each period. This resulted in 4 treatment combinations: TN-CON, TN-SBO, HS-CON, and HS-SBO. When goats were switched from TN to HS conditions, a transition period of 2 d was allowed (1 d at 25 °C, 1 d at 30 °C), but the change from HS to TN was abrupt. Goats had a 3-week pre-experimental period under TN conditions for adaptation to the diet and metabolic cages.

Goats were in 2 adjacent rooms with identical management conditions. Throughout the experiment (January to April), the room temperature for TN goats was maintained using electric heaters (3.5 kW; General Electric, Barcelona, Spain) equipped with a thermostat. The room of HS goats was provided with a temperature and humidity controlling system (Carel Controls Ibérica, Barcelona, Spain). A continuous 90 m^3^/h air turnover was maintained throughout the experiment.

Data of ambient temperature and humidity were recorded every 10 min throughout the experiment by a data logger (Opus 10, Lufft, Fellbach, Germany) and temperature-humidity index (THI) values were calculated according to NRC [18]: THI = (1.8 × T_db_ + 32) − [(0.55 − 0.0055 × RH) × (1.8 × T_db_ − 26.8)], where T_db_ is the dry bulb temperature (°C) and RH is the relative humidity (%). The THI values for TN varied between 59 and 65 throughout the day, whereas for HS, the THI values were 85 during the day and 77 during the night.

The total mixed rations for control and SBO-supplemented goats were fed ad libitum and formulated according to INRA [19]. Ingredients, chemical composition, and nutritive value of the rations are shown in Table 1. Drinking water was freely available at room temperature. Feed was offered once daily at 09:00 h at 115% of expected intake.

Goats were milked once daily throughout the experiment at 08:00 h with a portable milking machine (Westfalia-separator Ibérica, Granollers, Spain). Milking was conducted at a vacuum pressure of 42 kPa, a pulsation rate of 90 pulses/min, and a pulsation ratio of 66%. The milking routine included cluster attachment without udder preparation or teat cleaning, machine milking, machine stripping before cluster removal, and teat dipping in an iodine solution (P3-ioshield, Ecolab Hispano-Portuguesa, Barcelona, Spain).

### 2.2. Sample Collection, Analyses, and Measurements

Rectal temperatures and respiratory rates were recorded at 08:00, 12:00, and 17:00 h. The rectal temperature was measured by a digital clinical thermometer (Model ICO Technology “mini color” Barcelona, Spain; 32 to 43.9 ± 0.1 °C). The respiratory rate was measured by counting the inhalations and exhalations for 60 s with the aid of a digital chronometer (Model 900400; Deltalab, Barcelona, Spain).

Goats were weighted in 2 consecutive days at the start and end of each experimental period using a digital scale (Tru-Test AG500 Digital Indicator, Auckland, New Zealand; accuracy ± 20 g) to measure the change in BW. Additionally, BW values were used to calculate net energy balance using the following equation: energy balance = net energy intake − (NE_M_ + NE_L_).

Net energy for maintenance (NE_M_) was calculated using the following equation: NE_M_ = (0.0406 × BW^0.75^) according to INRA [19]. Maintenance costs were increased by 30% for HS goats as recommended by NRC [20]. Net energy for lactation (NE_L_) was calculated by using the following equation: NE_L_ = milk yield × [0.389 + 0.0052 (fat, g/kg − 35) + 0.0029 × (protein, g/kg − 31)] according to INRA [19].

Feed offer, feed orts, and water consumption (accuracy: ±20 g) were recorded daily throughout the measurement period, and samples of feed and orts were collected. For the determination of the digestibility coefficients, feces of each goat were daily collected and 10% of fresh feces were dried at 60 °C for 48 h. Then a composted sample (orts and feces) for each goat was stored in a refrigerated chamber until analysis. For the calculation of N retention, urine was collected in containers with 20 mL of H_2_SO_4_ (96%) and urine volume was daily measured (accuracy: ±2 mL). Urine samples (5% of total volume) were composted and stored at −25 °C for N content analysis. Feed, orts, and feces samples were ground through a 1-mm stainless steel screen, and then analyzed according to analytical standard methods [21]. The Dumas method (Leco analyzer, LECO Corp., St. Joseph, MI, USA) was used for N determinations and crude protein was calculated as percentage of N × 6.25.

Milk yield of each goat was recorded daily throughout the 5-days measurement period. A milk sample of approximately 50 mL was collected for two consecutive days during the measurement days of each period and preserved with an antimicrobial tablet (Bronopol, Broad Spectrum Microtabs II, D&F Control Systems Inc., San Ramon, CA, USA) at 4 °C until analysis. Milk samples were analyzed for fat, protein, and lactose using medium infrared spectrophotometry (MilkoScan FT2, Foss, Hillerød, Denmark). Milk somatic cell count was determined using an automatic cell counter (Fossomatic 5000, Foss Electric, Hillerød, Denmark). Devices used for milk analyses were previously calibrated for goat milk. Yields of fat, protein, and lactose were calculated using the corresponding milk yields for each sampling.

Additional milk sample of approximately 50 mL was collected individually at the last day of each period for milk FA analysis. Fat was obtained by centrifugation at 6000× *g* for 30 min and frozen at −80 °C until the analysis of FA profile using gas chromatography method as described by Bouattour et al. [14]. Individual FA methyl esters were identified by comparison of retention times with known FA methyl esters standards.

Blood samples were collected at the last day of each experimental period from the jugular vein into vacutainers (Venoject, Leuven, Belgium) before milking and feeding. Plasma was obtained by centrifugation of blood for 15 min at 1500× *g*, and stored at −20 °C for the analysis of non-esterified fatty acids (NEFA), and β-hydroxybutyrate. The NEFA were analyzed by the colorimetric enzymatic test ACS-ACOD method using a commercial kit (Wako Chemicals, Neuss, Germany). The β-hydroxybutyrate was determined by kinetic enzymatic method using commercial kit (Ranbut, Randox, Crumlin, UK). Furthermore, whole blood without anticoagulants was collected by insulin syringes and a single drop was immediately applied to disposable cartridges (iSTAT EC8^+^ cartridges, Abbott Point of Care Inc., Princeton, NJ, USA). Then, the cartridge was inserted into an i-STAT handheld analyzer maintained at room temperature (22 °C), and the results of electrolytes, hematocrit, hemoglobin, glucose, and urea were obtained.

### 2.3. Statistical Analyses

Data were analyzed by the PROC MIXED for repeated measurements of SAS v. 9.1.3 (SAS Institute Inc., Cary, NC, USA). The statistical mixed model contained the fixed effects of the temperature (TN and HS), dietary supplementation (CON and SBO), measurement day (day 1 to 5), period (1 to 4); the random effect of the animal; the interactions of temperature × supplementation, temperature × period, supplementation × period; and the residual error. For the data of rectal temperature and respiratory rate measured at 09:00, 12:00, and 17:00 h, a fixed factor of the hour of day was added to the model. For the data of digestibility, blood metabolites and changes of BW, the PROC MIXED was used without repeated measures, and consequently the measurement day effect was removed from the model. Data were tested for normality by evaluating the Shapiro–Wilk statistic using PROC UNIVARIATE of SAS. Data were transformed and the GROUP option in the REPEATED statement was used to separate variances and adjusted for unequal variances if needed. Differences between least square means were determined with the PDIFF test of SAS. Significance was declared at *p* < 0.05 and tendency at *p* < 0.10 unless otherwise indicated.

## 3. Results and Discussion

### 3.1. Rectal Temperature and Respiratory Rate

Heat-stressed goats showed increased (*p* < 0.001) average rectal temperatures (+0.99 °C) and respiratory rates (+77 breaths/min) compared to TN goats (Table 2). These results agree with what observed in the same goat breed exposed to similar conditions of HS [6,7]. The increase in respiratory rate in heat-stressed goats was for dissipating heat load by pulmonary water evaporation (HS goats drank greater amounts of water as indicated hereafter).

The supplementation with SBO had no effect on rectal temperature or respiratory rate in our goats. Similarly, feeding heat-stressed dairy cows with diets containing 5 to 7% fat has no effects on rectal temperature values [22]. Nevertheless, Gaughan and Mader [23] reported increased body temperature and respiratory rate in finishing steers exposed to hot and fed 5% of soybean oil. In contrast, crossbred steers fed diets containing 9% fat during hot weather experience 0.3 to 0.4 °C lower body temperatures compared to steers fed with 2.5% fat, suggesting less heat production in those steers fed high fat [24].

### 3.2. Body Weight Change, Feed Intake, and Energy Balance

On average, DM intake decreased (*p* < 0.001) by 37% in HS compared to TN goats (Table 2). The DM intake losses observed in the current study are similar to the values (−30 to −33%) observed in heat-stressed goats of the same breed in similar stage of lactation [6,7], but greater than losses (−21%) in late lactating goats [25]. Reducing feed intake is a mechanism to decrease heat production in HS conditions since heat increment of feeding in ruminants is a significant source of heat production [26]. The HS goats increased (*p* < 0.001) water consumption by 83% on average compared to TN goats. Increased water intake in HS conditions is mainly used for boosting heat loss by evaporation from the skin (sweating) and by respiration (panting) as previously reported in dairy goats [6,7,25].

Heat-stressed goats lost 104 g/d of BW, whereas TN goats gained 163 g/d. The HS goats were in negative energy balance (−0.60 Mcal/d), whereas TN goats were in positive energy balance (+0.91 Mcal/d), which could explain the changes observed in BW. The negative energy balance of HS goats was caused by the reduced DM intake and the increment in energy requirements for heat dissipation. Similar to dairy goats, heat-stressed dairy cows typically suffer negative energy balance [1]. It worth mentioning that a portion of the changes in BW of TN and HS goats included the inevitable variations in the digestive tract content, which were unknown in our study.

Supplementation with SBO did not affect DM intake or water consumption (Table 2), which agrees with the results obtained when SBO was supplemented (2 to 5%) to dairy goats [14,15] and cows [27]. Generally, DM intake is usually affected when high levels of lipids or strong flavor sources (e.g., fish oil) are added to the diet. In the current study, SBO level (4% of total DM) did not cause adverse effect on feed intake.

Feed supplementation with SBO had no effect on BW change. Liu et al. [27] reported that the dairy cows supplemented with 2.5% SBO partition more energy toward body tissue gain rather than milk synthesis. In our goats, SBO did not affect BW change, but increased milk fat content and consequently milk energy content output as discussed hereafter. Specie difference may explain the difference in response to SBO between dairy cows and goats since SBO causes milk fat depression in cows, but increases milk fat in goats [14,15].

### 3.3. Milk Yield and Milk Composition

Heat-stressed goats produced lower (*p* < 0.05) milk yield (−9%) and fat-corrected milk (−12%) than TN goats (Table 2). Nevertheless, late lactating goats of the same breed suffered no losses in milk yield under HS conditions [25]. It seems that the response of milk yield to HS varies according to lactation stage, with goats at earlier stages (e.g., current study) experiencing greater milk yield losses.

Heat stress decreased milk fat content by 6% compared to TN conditions, but this difference was not significant (*p* = 0.139). Furthermore, HS decreased (*p* < 0.001) protein content in milk by 15% as previously observed in dairy goats [5,6,7,25]. This decrease in milk protein could be partially explained by the increased sweat secretion that contains protein and urea [28] together with decreased protein intake under HS, which might have limited the availability of amino acids for milk protein synthesis [29]. Yields of milk fat, protein, and lactose were also depressed (*p* < 0.05) by HS (Table 2). Although mammary immunity is hindered by HS [30], heat load in the current study had no significant effect on milk somatic cell count, which is in accordance with the results obtained in dairy goats [6,7] and sheep [31].

No significant interaction between HS and SBO was detected for milk yield or milk composition (Table 2), indicating that response to SBO supplementation did not vary according to the ambient temperature. The supplementation with SBO did not modify milk yield, which agrees with what has been observed in dairy goats [14], sheep [32], and cows [27]. Mele et al. [15] detected an increase in milk yield of Saanen dairy goats when 4% SBO is added to a diet containing 63% forage, but milk yield is not affected in the low forage diet. The forage level in the current study (60%) was similar to the level used by Mele et al. [15], but differences in forage type, concentrate ingredients, and goat breed may explain the discrepancy between results. It worth mentioning that goats used in the present study are medium-yielding breed, and a greater effect of SBO on milk production might be expected in case of high-yielding breeds (e.g., Saanen).

The addition of SBO led to an increment in milk fat contents (+29%) and milk fat yields (+32%) on average. Increased milk fat content has been reported [14,15] when dairy goats are supplemented with SBO. In contrast, studies in dairy cows [27,33] reported a decrease in milk fat content of dairy cows supplemented with 2.5 to 5.0% SBO. Other studies reported no change in milk fat content by SBO in dairy cows [34] and ewes [32]. These contradictive results could be due to differences in specie, breed, physiological state, roughage source, and roughage: concentrate ratio in the diet. Compared with dairy cows, dairy goats are considered to be less sensitive to milk fat depression factors when vegetable oils are added to the diet [35]. Addition of SBO under TN or HS conditions increased milk fat without any negative effect on milk protein or lactose contents (Table 2). This result agrees with what obtained by others when diet was supplemented with SBO in dairy goats [14,15], sheep [32], and cows [33,34].

Given the significant increment in milk fat content observed in the present study, sale price of milk would increase in a quality-based milk payment system. Consequently, profits resulting from milk sales would increase, especially if SBO is accessible at reasonable prices. In the current Spanish market, SBO price is €912/t, and goat milk is paid at the rate of €0.086/cheese extract unit (fat% + protein%). Taking the average values reported in Table 2 for TN and HS goats, cheese extract increased from 7.06 to 8.25% in CON and SBO goats, respectively. Consequently, milk price is €0.604 and €0.705/kg milk in CON and SBO goats, respectively. Thus, milk income per goat is €1.105 and €1.318 for CON and SBO goats, respectively (average milk yield was 1.83 and 1.87 for CON and SBO goats, respectively). Given that the cost of SBO feeding is €0.066/goat on average, the increment in milk income (€0.213/goat) results in net profit of €0.147/goat/day.

### 3.4. Milk Fatty Acid Profile

Data of milk FA profile as affected by HS and SBO supplementation are shown in Table 3. Heat stress decreased (*p* < 0.001) de novo (< C16) and mixed (C16) FA, but increased (*p* < 0.001) preformed FA (> C16) contents. Additionally, HS decreased (*p* < 0.001) the concentrations of saturated FA and increased (*p* < 0.001) monounsaturated FA concentrations with no effect on the polyunsaturated FA. Considering data of milk fat yield (Table 2) and milk FA contents (Table 3), calculated FA yields corrected according to Glasser et al. [36] were: < C16, 30.4 g/d; C16, 29.1 g/d; > C16, 19.1 g/d for TN goats, and < C16, 21.5 g/d; C16, 20.1 g/d; > C16, 23.9 g/d for HS goats. Thus, because of HS the production of totally (< C16) or partially (C16) de novo FA was reduced (*p* < 0.01) by 16.4 g/d, whereas FA extracted from blood did not change (only +4.7 g/d; *p* > 0.10). Since glucose is an important substrate for supporting the synthesis of de novo FA [37], depressed de novo FA synthesis in the current study by HS would increase glucose availability for lactose synthesis, with a concomitant increase in milk yield. Nevertheless, both lactose yield and milk yield were reduced (*p* < 0.05) by HS.

Desaturase indices decreased (C16 and CLA indices; *p* < 0.05) or tended to decrease (C14 index; *p* < 0.10) by HS (Table 3). These indices represent a proxy for the Δ9-desaturase in the mammary gland [39], and research in dairy goats demonstrated a positive correlation between these FA ratios and the activity and the gene expression of Δ9-desaturase [35]. Our results indicate that the activity of Δ9-desaturase decreased by HS. However, Liu et al. [40] reported no change in the desaturation activity by HS in dairy cows.

Supplementation with SBO decreased (*p* < 0.001) the concentration of de novo and mixed FA, but increased (*p* < 0.001) the preformed FA. Mele et al. [15] also observed that goats fed high forage diet (63%, which is similar to the current study) experience depressed short- and medium-chain FA when SBO was supplemented (4%). The increase in long chain FA has an inhibitor effect on de novo FA synthesis in the mammary gland [41], which resulted in lower concentrations of short- and medium-chain FA in SBO goats. Interaction between HS and SBO for de novo FA tended to be significant (*p* = 0.0.93) since the reduction in milk de novo FA caused by SBO was greater under HS (−30%) compared with TN (−20%) conditions.

Feeding SBO sharply increased (*p* < 0.001) milk C18:0 and C18:1, which might be related to the biohydrogenation of FA from SBO in the rumen to C18:0 and *trans*-C18:1. The increase in milk C18 FA was compensated for by the decrease (*p* < 0.001) in milk C16:0 and most of the de novo FA. The increase in long chain FA, when SBO was supplemented, could be related to the increment in blood NEFA levels by more than 50% on average (see hereafter). These NEFA are taken up by the mammary gland and used for milk fat synthesis. The situation in case of HS is totally different as no increase in blood NEFA values was observed (see later) to justify the increase in long chain FA.

Out of the 3 desaturation indices calculated in the current study, the CLA index was the only one that was reduced by SBO supplementation. Reduced desaturation activity by SBO feeding has been reported in dairy goats [15], although another study [14] found no change. Chilliard et al. [41] pointed out that Δ9-desaturase indexes are generally lower in goats compared with cows for medium-chain FA, but not for C18 FA, which might indicate a specie-dependent response of Δ9-desaturase activity according to chain length.

The SBO supplementation dramatically increased CLA content in milk of dairy goats (Table 3) because of the increment in *trans*-vaccenic acid (TVA). This result is similar to what previously reported in dairy goats [14,15,16,17] and ewes [16,32]. The effect of SBO on TVA and CLA contents was similar in TN and HS conditions (+650 and +395% increments in TVA and CLA concentrations, respectively, on average). There is a strong positive correlation between CLA and TVA levels in milk of dairy goats [15,41] since TVA is desaturated in the mammary gland to CLA. In fact, linoleic acid is the predominant FA in SBO and is an important source of TVA production in the rumen [39].

Odd FA (C11:0, C13:0, C15:0, and C17:0) are predominantly originated from rumen micro-organisms lipids in addition to small amounts from de novo synthesis from propionate in mammary cells [42]. In the current study, the concentration of these four odd FA were reduced (*p* < 0.001) by SBO supplementation. Heat stress also decreased (*p* < 0.01) C11:0, C13:0, and C15:0, but C17:0 was increased (*p* < 0.01). Similarly, triacylglycerol groups containing FA with odd number of carbons show a significant reduction in heat-stressed dairy cows [40]. Milk C15:0 and C17:0 are negatively related to rumen acetate production [43]. Thus, it seems that both HS and SBO altered rumen fermentation and ruminal microbiota. Profiling the rumen microbiota by 16sRNA gene cloning confirmed that HS induces significant changes in microbial diversity in heifers [44]. Additionally, we previously showed that HS goats experience lower mean daily rumen pH than TN goats despite that fact that both groups eat the same amount of feed [45]. Further, infusing SBO results in a decrease in ruminal pH in beef heifers [46]. These alterations in rumen pH by HS and SBO could affect rumen fermentation and the microbial population, which could explain the reduction in milk odd FA concentrations. There was HS by SBO interaction (*p* < 0.05) for milk C17:0 since the reduction caused by SBO was more marked in HS (−39%) than in TN conditions (−22%).

From the point of view of human health, HS and SBO reduced milk atherogenicity index by −32 and −54%, respectively (Table 3). When the effects of HS and SBO were jointed (i.e., HS-SBO goats), the atherogenicity index was dramatically reduced by −70% compared to the control (i.e., TN-CON goats). Nevertheless, there is little evidence of an atherogenic effect of saturated FA (C12:0, C14:0, and C16:0), and that they could even have protective effect compared to low-fat, high-carbohydrate diets [41]. Therefore, the reduction in this this index by SBO supplementation could be beneficial for human health only in cases in which there is excessive fat consumption. Furthermore, milk of HS and SBO goats had greater contents of monounsaturated FA (Table 3). The mono-unsaturated FA are advantageous as they increase the concentration of high-density lipoproteins that prevent cholesterol from accumulation on blood vessel walls and transport it to the liver [47]. Specifically, TVA is the major *trans* C18:1 in milk and has a protective role in cardiovascular diseases [48]. Additionally, CLA may have an anti-carcinogenic effect as demonstrated in vivo by using animal models [49], where supplementation with *cis*-9, *trans*-11 C18:2 CLA reduces tumorigenesis. Although HS apparently results in a healthier milk FA profile, it negatively affects milk protein, and could impair milk coagulation properties and reduce cheese yield in dairy goats [29].

### 3.5. Digestibility and Nitrogen Retention

Digestibility coefficient values of DM, crude protein, neutral detergent fiber, and acid detergent fiber, as well as N retention, are shown in Table 4. Heat stress increased (*p* < 0.01) digestibility coefficients by 5 to 9 points. The acid detergent fiber digestibility improvement was the highest (+9 points) followed by neutral detergent fiber digestibility (+7 points). This increment in digestibility caused by HS is greater than the observed in the same goat breed at late lactation in HS conditions [25]. Similarly, greater digestibility by HS has been observed in male goats [50] and heifers [51]. The increased digestibility under HS conditions might be partially due to the reduction in feed intake. Another reason for the enhanced digestibility under HS conditions could be a depressed passage rate of the solid phase of digesta as indicated by Bernabucci et al. [51] and Salama et al. [2]. Nitrogen intake and N retention decreased by 38 and 30%, respectively, in HS goats, which is related to the reduced feed intake. In contrast, we observed in a previous study that HS had no effect on N retention in late lactation dairy goats, even with less N intake [25]. Lower retained N could partially explain the reduction in milk protein yield in HS goats.

Despite the positive effect on milk fat and milk FA profile, the SBO supplementation did not affect the digestibility or N retention of dairy goats (*p* > 0.10). This result could indicate normal digestion process when SBO was supplemented. Almeida et al. [52] also found no effect of SBO supplementation (2% on DM basis) on diet digestibility in Saanen dairy goats. The DM intake in the present study was not affected by SBO (Table 2), which might partially explain the absence of significant effect on digestibility.

### 3.6. Blood Metabolites

Data of blood indicators in TN and HS goats supplemented or not with SBO are shown in Table 5. Heat stress decreased (*p* < 0.05) or tended (*p* < 0.10) to decrease Na, K, total CO_2_, CO_2_ partial pressure, HCO_3_, base excess, anion gap, and urea concentrations in the blood of goats. However, HS had no effect on glucose, pH, Cl, hematocrit, or hemoglobin values. The deceased CO_2_ partial pressure and HCO_3_ under HS conditions agree with results reported in dairy goats [25]. The greater respiration rate observed in HS goats contributed to greater loss of CO_2_ and lowering the carbonic acid content of the blood. To maintain the blood pH constant, HCO_3_ is transferred from blood to urine by the kidney. The decrease in blood HCO_3_ (−5 points approximately) is similar to what has been reported in heat-stressed dairy goats [25]. Blood K tended to be greater (*p* = 0.057) in HS goats compared to TN goats. Heat-stressed dairy cows increase their requirements of K since sweat contains high concentrations of K [53]. The increased blood K in HS goats is presumably occurred to meet K requirements although DM intake (and consequently mineral intake) was reduced. However, goats in the present experiment had available mineral-vitamin blocks at their choice, which helped them to get their mineral needs.

Although DM intake decreased by HS (Table 2), HS goats had similar blood glucose levels as TN goats. This result confirms the previous data obtained in heat-stressed dairy goats [6,7,25] and ewes [31], where DM intake is depressed without changes in blood glucose. In the current study, the saved glucose that was caused by lower de novo FA synthesis might partially explain the no variation in blood glucose level between TN and HS goats. In addition, HS goats [29] and ewes [31] have been reported to secrete lower insulin in response to glucose infusion compared to TN animals, which might explain the ability of HS goats to keep similar glucose levels as TN animals. The reduction (*p* < 0.001) in blood urea concentration by HS could be related to the decreased DM intake and, consequently, reduced N intake. Although HS goats suffered negative energy balance, blood NEFA values did not increase. The fact that lipid tissue becomes more resistant to lipolytic signals in HS conditions has been observed in dairy goats [29], sheep [31], and cows [1].

Soybean oil supplementation decreased (*p* < 0.05) blood pH. This reduction in blood pH, albeit significant, is of low physiological importance since blood pH is regulated by a complex system of buffers that continuously work to maintain it slightly basic in a range of 7.35 to 7.45 in most mammals [54]. Blood pH values in the current experiment were within this normal range. Supplementation with SBO increased blood NEFA concentration, which agrees with previous results in dairy cows [27]. This increase in NEFA concentration was not accompanied by an increment in β-hydroxybutyrate levels, which might indicate that NEFA were rapidly taken up by the mammary gland for fat synthesis and were not converted to ketone bodies in the liver.

## 4. Conclusions

Heat stress caused losses in milk yield and milk components in dairy goats. Heat stress additionally altered milk fatty acid profile, which is featured by a decrease in saturated fatty acids and an increase in monosaturated fatty acids. Feeding soybean oil incremented milk cheese extract, which would increase profits in quality-based milk payment system. Soybean oil supplementation increased the percentage of long chain fatty acids in milk, and decreased the de novo and saturated fatty acids. The supplementation with 4% soybean oil increased milk fat, trans-vaccenic acid and conjugated linoleic acid (*cis*-9, *trans*-11 isomer). There was no interaction between oil supplementation and heat stress for most of the studied variables, indicating that dairy goats responded to soybean oil in a similar manner, regardless the ambient temperature.

## Figures and Tables

**Table 1:** Ingredients and nutrient composition of the control (CON) diet without supplementation and the supplemented diet with 4% sobybean oil (SBO). Values are expressed on dry matter basis.

Item	CON	SBO
Ingredient, %		
Alfalfa hay	60.4	60.4
Barley, ground	15.0	11.0
Soybean oil	—	4.0
Beet pulp	9.1	9.1
Corn, ground	7.5	7.5
Soybean meal	3.0	3.0
Sunflower meal	3.0	3.0
Molasses	1.0	1.0
Salt	0.6	0.6
Sodium bicarbonate	0.2	0.2
Vitamin-mineral complex	0.2	0.2
Component, %		
Dry matter ^1^	89.7	89.9
Crude protein	17.2	16.8
Ether extract	2.07	5.88
Neutral detergent fiber	34.7	33.5
Acid detergent fiber	19.9	19.6
Nutritive value ^2^		
UFL, ^3^ /kg	0.86	0.95
NE_L_, Mcal/kg	1.51	1.68
PDI, ^4^ g/kg	82.3	78.9
PDIA, ^5^ g/kg	40.8	39.7
Calcium, g/kg	7.22	7.18
Phosphorous, g/kg	2.68	2.52

^1^ Dry matter was expressed as a percentage of as fed. ^2^ Calculated according to the INRA [19]. ^3^ Forage unit for lactation (1 UFL = 1.76 Mcal of NE_L_). ^4^ Protein digestible in the intestine from dietary and microbial origin. ^5^ Protein digestible in the intestine from dietary origin.

**Table 2:** Least squares means for physiological variables, feed intake, and milk production in dairy goats under thermoneutral (TN) and heat stress (HS) conditions. In each ambient temperature goats were fed a control diet (CON) or supplemented with 4% soybean oil (SBO) ^1^.

Variable	TN		HS		SEM	Effect ^2^ (*p*<)		
	CON (*n* = 8)	SBO (*n* = 8)	CON (*n* = 8)	SBO (*n* = 8)		T	S	T × S
Rectal temperature, °C	38.63	38.67	39.57	39.70	0.08	0.001	0.611	0.495
Respiratory rate, breaths/min	34	35	110	114	4	0.001	0.733	0.678
Body weight change, kg	3.49	3.36	−2.08	−2.28	0.97	0.001	0.597	0.745
Dry matter intake, kg/d	2.28	2.26	1.49	1.34	0.09	0.001	0.495	0.483
Energy balance, Mcal/d	0.86	0.95	−0.53	−0.67	0.11	0.001	0.829	0.300
Water consumption, L/d	6.14	6.28	10.63	12.06	1.04	0.001	0.310	0.480
Milk yield, kg/d	1.88	1.99	1.78	1.75	0.11	0.013	0.606	0.230
Fat-corrected milk, kg/d ^3^	2.18	2.31	1.84	2.13	0.13	0.004	0.035	0.560
Milk composition, %								
Fat	4.08	5.17	3.75	4.95	0.21	0.139	0.001	0.775
Protein	3.42	3.41	2.87	2.97	0.10	0.001	0.560	0.569
Lactose	4.35	4.51	4.15	4.28	0.05	0.001	0.007	0.791
Fat yield, g/d	74	100	65	84	6	0.026	0.003	0.510
Protein yield, g/d	60	64	48	48	3	0.001	0.491	0.556
Lactose yield, g/d	76	84	68	68	5	0.011	0.386	0.385
Somatic cell count, Log_10_	5.54	5.57	5.67	5.63	0.20	0.456	0.711	0.587

^1^ Each goat received one treatment (TN-CON, TN-SBO, HS-CON, HS-SBO) in one of the 4 experimental period (14 days for adaptation and 5 days for measurements). The data shown for each treatment are the average of the experimental days of the 4 periods. ^2^ Effects of ambient temperature (T), supplementation (S), and their interaction (T × S). ^3^ Fat corrected milk at 3.5%; fat-corrected milk = kg of milk yield × [0.432 + 0.162 × (fat %)].

**Table 3:** Least squares means for milk fatty acids (FA) expressed as % of total FA methyl esters in dairy goats under thermoneutral (TN) and heat stress (HS) conditions. In each ambient temperature goats were fed a control diet (CON) or supplemented with 4% soybean oil (SBO) ^1^.

Variable	TN		HS		SEM	Effect ^2^ (*p*=)		
	CON (*n* = 8)	SBO (*n* = 8)	CON (*n* = 8)	SBO (*n* = 8)		T	S	T × S
C4:0	1.20	1.22	1.19	1.21	0.04	0.616	0.364	0.762
C6:0	2.02	2.17	1.94	2.14	0.11	0.462	0.042	0.771
C8:0	2.61	2.81	2.68	2.53	0.17	0.451	0.856	0.224
C10:0	11.45	9.79	10.70	7.73	0.60	0.005	0.001	0.140
C11:0	0.35	0.30	0.28	0.23	0.02	0.001	0.001	0.912
C12:0	6.91	4.24	5.74	2.80	0.51	0.005	0.001	0.724
C13:0	0.23	0.19	0.19	0.14	0.01	0.001	0.001	0.378
C14:0	12.94	9.71	11.94	7.50	0.69	0.011	0.001	0.279
C14:1	0.27	0.15	0.15	0.08	0.05	0.006	0.008	0.399
C15:0	0.94	0.68	0.79	0.58	0.05	0.003	0.001	0.589
C16:0	38.23	25.68	30.59	22.11	1.81	0.001	0.001	0.154
C16:1	1.00	0.60	0.63	0.44	0.19	0.028	0.018	0.335
C17:0	0.55	0.43	0.75	0.46	0.06	0.008	0.001	0.033
C18:0	4.87	11.80	9.07	17.29	1.73	0.003	0.001	0.622
*Trans*-9 C18:1	0.14	0.56	0.15	0.65	0.04	0.095	0.001	0.225
*Trans*-11 C18:1 (TVA ^3^)	0.68	4.76	0.71	5.68	1.49	0.614	0.001	0.640
*Cis*-9 C18:1	12.58	19.59	18.84	23.00	1.29	0.001	0.001	0.183
*Cis*-11 C18:1	0.37	0.78	0.54	0.97	0.05	0.001	0.001	0.775
C18:2n6t	0.18	0.42	0.17	0.43	0.04	0.968	0.001	0.840
C18:2n6c	2.48	2.55	2.90	2.67	0.17	0.045	0.529	0.226
C20:0	0.15	0.22	0.18	0.26	0.01	0.023	0.001	0.536
C18:3n3 + C20:1	0.69	0.55	0.72	0.50	0.05	0.733	0.001	0.315
*Cis*-9, *trans*-11 C18:2 (CLA ^4^)	0.47	2.17	0.37	1.95	0.62	0.685	0.001	0.875
C22:0	0.05	0.09	0.05	0.11	0.02	0.719	0.004	0.591
C20:4n6	0.15	0.10	0.23	0.13	0.02	0.003	0.001	0.840
Saturated FA	80.95	67.76	74.52	63.5	1.32	0.001	0.001	0.381
Mono-unsaturated FA	15.03	26.46	21.01	30.82	1.27	0.001	0.001	0.399
Poly-unsaturated FA	3.95	5.79	4.37	5.68	0.45	0.736	0.005	0.565
De novo FA ^5^	38.54	30.90	35.22	24.66	1.02	0.001	0.001	0.093
Mixed FA ^5^	39.23	26.28	31.21	22.56	1.81	0.001	0.001	0.140
Preformed FA ^5^	22.87	43.60	34.00	53.64	2.06	0.001	0.001	0.703
Elongase ^6^	30.87	54.28	47.17	63.93	3.07	0.001	0.001	0.169
Atherogenicity index ^7^	5.26	2.29	3.40	1.60	0.29	0.001	0.001	0.015
Δ9-Desaturase index ^8^								
C14	0.020	0.015	0.013	0.010	0.006	0.096	0.300	0.724
C16	0.025	0.023	0.020	0.018	0.003	0.049	0.295	1.000
CLA ^4^	0.40	0.31	0.34	0.25	0.040	0.039	0.004	0.885

^1^ Each goat received one treatment (TN-CON, TN-SBO, HS-CON, HS-SBO) in one of the 4 experimental periods (14 days for adaptation and 5 days for measurements). The data shown for each treatment are the average of the experimental days of the 4 periods. ^2^ Effects of ambient temperature (T), supplementation (S), and their interaction (T × S). ^3^ Trans vaccenic acid. ^4^ Conjugated linoleic acid. ^5^ De novo = milk FA < 16 carbons in length; mixed = milk FA 16 carbons in length; preformed = milk FA > 16 carbons in length. ^6^ Elongation of C16 to C18 calculated as (C18 + C18:1)/(C16 + C16:1 + C18 + C18:1) ×100. ^7^ Atherogenicity index calculated according to Ulbricht and Southgate [38] as: (C12:0 + 4 × C14:0 + C16:0)/(mono-unsaturated FA + poly-unsaturated FA). ^8^ Calculated for each pair of FA as (product of Δ9-desaturase)/(product of Δ9-desaturase + substrate of Δ9-desaturase); e.g., C14:C14:1/(C14:1 + C14:0).

**Table 4:** Least squares means for digestibility coefficients and nitrogen retention of dairy goats under thermoneutral (TN) and heat stress (HS) conditions. In each ambient temperature, goats were fed a control diet (CON) or supplemented with 4% soybean oil (SBO) ^1^.

Variable	TN		HS		SEM	Effect ^2^ (*p*=)		
	CON (*n* = 8)	SBO (*n* = 8)	CON (*n* = 8)	SBO (*n* = 8)		T	S	T × S
Digestibility, %								
Dry matter	67.8	68.5	74.0	72.6	1.4	0.001	0.778	0.455
Organic matter	68.9	69.6	75.1	73.9	1.3	0.001	0.850	0.469
Crude protein	73.4	74.7	78.8	78.6	1.3	0.001	0.654	0.559
Neutral detergent fiber	50.5	50.2	58.1	56.6	2.4	0.007	0.708	0.804
Acid detergent fiber	43.5	43.6	52.2	52.8	2.9	0.004	0.941	0.989
Apparent absorption, %								
Nitrogen retention, g/d	21.8	20.2	13.7	15.5	2.3	0.009	0.951	0.454

^1^ Each goat received one treatment (TN-CON, TN-SBO, HS-CON, HS-SBO) in one of the 4 experimental period (14 days for adaptation and 5 days for measurements). The data shown for each treatment are the average of the experimental days of the 4 periods. ^2^ Effects of ambient temperature (T), supplementation (S), and their interaction (T × S).

**Table 5:** Least squares means for blood metabolites in dairy goats under thermoneutral (TN) and heat stress (HS) conditions. In each ambient temperature, goats were fed a control diet (CON) or supplemented with soybean oil (SBO) ^1^.

Variable	TN		HS		SEM	Effect ^2^ (*p*=)		
	CON (*n* = 8)	SBO (*n* = 8)	CON (*n* = 8)	SBO (*n* = 8)		T	S	T × S
pH	7.46	7.45	7.47	7.44	0.01	0.511	0.038	0.205
Na, mmol/L	152	151	147	147	1	0.001	0.790	0.710
K, mmol/L	3.65	3.69	3.94	3.83	0.11	0.057	0.729	0.489
Cl, mmol/L	110	112	110	113	2	0.777	0.106	0.777
Total CO_2_, mmol/L	28.6	27.1	22.9	21.3	1.0	0.001	0.129	0.951
CO_2_ partial pressure, mmHg	39.0	37.8	29.7	29.7	1.6	0.001	0.711	0.692
HCO_3_, mmol/L	27.3	26.0	22.0	20.3	1.0	0.001	0.146	0.852
Anion gap	17.0	16.9	18.8	17.8	0.6	0.039	0.360	0.476
Base excess, mmol/L	3.25	1.75	−1.88	−3.88	1.03	0.001	0.102	0.811
Hematocrit, %PCV	18.1	17.6	18.1	17.3	0.8	0.821	0.410	0.821
Hemoglobin, g/dL	6.16	6.00	6.16	5.88	0.28	0.826	0.431	0.862
Glucose, mg/dL	55.1	53.8	56.4	55.4	1.6	0.374	0.462	0.907
Blood urea N, mg/dL	21.1	21.4	17.1	16.8	1.0	0.001	0.949	0.748
Non-esterified fatty acids, mmol/L	0.07	0.11	0.09	0.12	0.02	0.202	0.025	0.731
β-hydroxybutyrate, mmol/L	0.65	0.61	0.96	0.72	0.08	0.168	0.224	0.493

^1^ Each goat received one treatment (TN-CON, TN-SBO, HS-CON, HS-SBO) in one of the 4 experimental period (14 days for adaptation and 5 days for measurements). The data shown for each treatment are the average of the experimental days of the 4 periods. ^2^ Effects of ambient temperature (T), supplementation (S), and their interaction (T × S).

## Data Availability

Not applicable since no unpublished data were reported in the present study.
